# The Lost Art of Prescription Writing

**Published:** 1910-09

**Authors:** 


### THE LOST ART OF PRESCRIPTION WRITING.

EXTEMPORANEOUS PRESCRIPTION WRITING is well-nigh a lost art. There are several reasons for this,—one is, the subject is not thoroughly taught, as it should be, in the medical colleges, even materia medica and therapeutics being more or less neglected. The curriculum is so extensive and the terms of instruction so relatively short, there is so much to be learned in the four sessions that it is nearly impossible for the most earnest student to master it all; and the subject of materia medica being less attractive to the average student than surgery, obstetrics or bacteriology or practice, is skimmed over just enough to enable one to pass. The student has in reservation the hope that between the sessions, at home, he can study the physical and other properties of drugs, and read up on practical therapeutics. Besides this he is aware that there is available an infinite variety of ready made prescriptions, some most excellent ones, and it is very handy and less trouble to prescribe some of these proprietaries which have stood the test of time and experience in the hands of some of our ablest practitioners and teachers than to write a prescription.

But every physician should thoroughly understand the drugs of the materia medica; be acquainted with their therapeutic properties; know the incompatibles, and the affinities, and in case of the poisonous drugs, the antidotes. In other words, there is need of more and better instruction in the art of prescription writing, of combining drugs to meet certain indications.

To this end Dr. Geo. L. Servoss, of Fairview, Nevada, whose contributions on certain of the alkaloidal salts have appeared in the "Red Back" and been received with much favor, has formulated his extensive experience and observation as a practitioner and as a teacher of therapeutics in a mail quiz course, consisting of forty-six lessons. This course he now offers to the profession.

The first two lessons will be devoted to introductory features. Then will follow the consideration of the various classifications of drugs as to their actions in general, and these in turn by the consideration of diseases in general with the application of therapeutic agents. His fee for the entire course will be $35 regularly, but until October 1st he will make a special fee of $25 to all those who enroll prior to that date.

